# Impact of chronic kidney disease on health-related quality of life in adults: a systematic review and meta-analysis protocol

**DOI:** 10.3389/fneph.2025.1630718

**Published:** 2025-10-27

**Authors:** William Wilberforce Amoah, Chikaodili Ihudiebube-Splendor, Victor Luckyboy Dzramado

**Affiliations:** ^1^ Department of Emergency and Critical Care Nursing, School of Nursing and Midwifery, Kwame Nkrumah University of Science and Technology, Kumasi, Ghana; ^2^ Department of Nursing Sciences, University of Nigeria, Nsukka, Nigeria; ^3^ Department of Health Information, Cape Coast Teaching Hospital, Cape Coast, Ghana

**Keywords:** chronic kidney disease, health-related Quality of Life (HR QoL) outcomes, impact, hypertension, mortality

## Abstract

**Introduction:**

Chronic Kidney Disease (CKD) significantly impacts patients’ health-related quality of life (HRQoL), yet comprehensive evidence synthesis remains limited, particularly from African contexts. This systematic review aims to evaluate how CKD affects HRQoL in adult patients and identify the most impacted domains across disease stages, providing evidence to guide patient-centered care and health policy.

**Methods:**

Following PRISMA-P 2020 guidelines, we will systematically search PubMed, Embase, Scopus, Web of Science, Cochrane Library, and grey literature for observational studies and clinical trials evaluating HRQoL in adults (≥18 years) with CKD using validated instruments (SF-36, KDQOL, EQ-5D). Two independent reviewers will conduct study selection, data extraction, and quality assessment using the Newcastle-Ottawa Scale and Cochrane Risk of Bias Tool. Meta-analysis will be performed where feasible, with subgroup analyses by CKD stage, treatment modality, and geographic region.

**Expected outcomes:**

This review will provide nurses and clinicians with comprehensive evidence on HRQoL impairments across CKD stages, inform development of targeted psychosocial interventions, and guide resource allocation for holistic patient care. Findings will support healthcare providers in addressing not only physiological parameters but also patients’ subjective wellbeing and quality of life.

**Systematic review registration:**

https://www.crd.york.ac.uk/PROSPERO/, identifier CRD420251036629.

## 1 Introduction

Chronic kidney disease (CKD) is a progressive condition associated with significant morbidity and mortality. Given its long-term nature, CKD affects various domains of health-related quality of life (HRQoL), including physical, psychological, and social well-being. Understanding the extent of HRQoL impairment in CKD patients can inform clinical interventions and healthcare policies.

Chronic kidney disease is a gradual loss of kidney function over time (1). It encompasses five stages based on the kidney’s ability to filter waste, measured by the glomerular filtration rate (GFR). CKD progresses through stages 1 to 5, with stage 1 being the mildest form (normal GFR but with signs of kidney damage) and stage 5 being the most severe (GFR less than 15 mL/min/1.73m2) (2). In early stages, CKD might be asymptomatic or cause mild symptoms like fatigue, swelling, or increased blood pressure (3). As CKD progresses, symptoms may worsen. Treatment focuses on slowing the progression through medications, lifestyle changes, and addressing the underlying causes, such as hypertension or diabetes. End-Stage Renal Disease (ESRD) is the final stage of Chronic Kidney Disease (stage 5 of CKD), where there is an irreversible loss of kidney function with a glomerular filtration rate (GFR) of less than 15ml/min/1.73m2 (4). At this stage, the kidneys cannot adequately filter waste, leading to life-threatening imbalances in fluids and electrolytes. ESRD causes more severe symptoms, including persistent fatigue, fluid overload, shortness of breath, and uremia (build-up of waste products) (3).

The worldwide prevalence of Chronic Kidney Disease (CKD) is 10-13% and is estimated to be increasing by approximately 8% annually (5). Persons living with CKD usually experience a diminished quality of life, an increased risk for cardiovascular diseases and a reduced life expectancy (6). The management of CKD has significant clinical, social, and financial consequences for patients, nurses, doctors, and the healthcare system (7). Persons with CKD require renal replacement therapy (dialysis or transplantation) (8–10). In 2019, 1.4 million patients were reported to be receiving renal replacement therapy (RRT) worldwide (11).

Chronic kidney disease is high in African countries with an estimated overall prevalence of 8%-16%, corresponding to nearly 500 million affected individuals, of whom 78% (387.5 million) are in low-to-middle-income countries (12). In Ghana, the prevalence of CKD is 13.3% with a mortality rate of 5% (5, 13), and an increase of CKD is reported at five times the rate of world population growth and is not expected to level out soon (13).

Patients with CKD confront a variety of challenges, including ongoing symptoms, difficult treatments, fluid restrictions, uncertainty about life, and a dependence on technology. Other challenges are dietary management, medication adherence and physical activity. These challenges have an impact on patients’ autonomy, especially once they begin receiving renal replacement therapy (9, 14, 15).

Most patients with CKD in Ghana and sub-Saharan Africa (SSA) are aged between 20 and 50 years representing the economically productive group (16, 17). The burden of end-stage renal disease in Ghana and sub-Saharan Africa is high (17, 18). In one study, 166 of 3317 patients admitted to hospital had renal disease, and of these, 45 (27.1%) died mainly from renal failure (18). Recent data from Korle-Bu Teaching Hospital in Ghana showed that 15% of all medical admissions have kidney disease. In addition, 10% of all deaths on the medical wards are due to end-stage renal disease (19).

Health-related quality of life (HRQoL) represents a multidimensional construct encompassing physical, psychological, and social domains of health from the patient’s perspective, extending beyond mere absence of disease. For patients with CKD, particularly those with ESRD, HRQoL assessment provides valuable insights into the comprehensive impact of the disease and its treatment on daily functioning and wellbeing. Multiple studies have demonstrated that patients with CKD experience significant impairments in HRQoL compared to the general population, with factors such as symptom burden, treatment modality, and psychosocial factors contributing to these differences.

A systematic review evaluating the impact of CKD on HRQoL would consolidate current knowledge, identify specific domains most affected by the disease, and provide an evidence base for developing targeted interventions. Such knowledge synthesis is particularly important given the growing burden of CKD globally and the need for comprehensive, patient-centered care approaches that address not only physiological parameters but also patients’ subjective experiences and overall quality of life.

### 1.1 Rationale

Despite growing recognition of HRQoL as a critical patient-centered outcome in CKD management, existing systematic reviews have important limitations. Previous reviews have predominantly focused on specific treatment modalities (dialysis or transplantation) rather than examining HRQoL comprehensively across all CKD stages. Additionally, most existing syntheses have been conducted in high-income countries, with limited representation of low- and middle-income settings, particularly African contexts where CKD burden is rising rapidly and healthcare resources differ substantially.

Furthermore, while several reviews have examined overall HRQoL scores, few have systematically analyzed which specific domains (physical, mental, or social functioning) are most severely affected at different disease stages. This granular understanding is essential for developing targeted interventions. There is also limited synthesis of how demographic and clinical factors—such as age, comorbidities, and socioeconomic status—moderate the relationship between CKD and HRQoL across diverse populations.

This knowledge gap hampers the development of evidence-based, targeted interventions to improve patients’ quality of life beyond physiological management. Our systematic review addresses these gaps by: (1) examining HRQoL across all CKD stages from early disease to end-stage renal disease; (2) including global evidence with particular attention to underrepresented regions; (3) systematically identifying which HRQoL domains are most affected; and (4) analyzing moderating factors that influence this relationship. Such comprehensive synthesis will provide valuable insights for healthcare providers, policymakers, and researchers aiming to enhance holistic care for patients with CKD.

### 1.2 Objective of the systematic review

The main objective is to evaluate the impact of CKD on HRQoL in the adult patients by analyzing findings from existing studies.

Specific Objectives.

1. To assess the impact of CKD on the HRQoL of affected patients.
2. To identify specific domains of HRQoL most affected by CKD, including physical health, mental health, and social functioning.
3. To determine differences in HRQoL between the different stages of CKD.
4. To assess the impact of dialysis and kidney transplantation on HRQoL.
5. To determine the influence of demographic and clinical factors (e.g., age, gender, comorbidities) on HRQoL in CKD patients.

## 2 Methods

This study protocol will follow the PRISMA-P (Preferred Reporting Items for Systematic Review and Meta-Analysis Protocols) guidelines. The systematic review will be conducted in accordance with these guidelines to ensure transparent and comprehensive reporting of the methodology employed, facilitating replication and critical evaluation of the review process.

### 2.1 Eligibility criteria

#### 2.1.1 Inclusion criteria

##### 2.1.1.1 Population

Adults (≥18 years) diagnosed with CKD at any stage, including those undergoing dialysis or with kidney transplants. The population will include patients across the spectrum of CKD severity, from early-stage disease (stages 1-3) to advanced disease (stages 4-5) and end-stage renal disease requiring renal replacement therapy. Both incident and prevalent cases of CKD will be considered, and there will be no restrictions based on demographic characteristics such as gender, ethnicity, or socioeconomic status.

##### 2.1.1.2 Intervention

Diagnosis of CKD. While this review focuses on a condition rather than a specific intervention, the “intervention” component in this context refers to the presence of CKD as the exposure of interest. Studies examining patients with CKD of any etiology will be eligible, including but not limited to diabetic nephropathy, hypertensive nephrosclerosis, glomerulonephritis, polycystic kidney disease, and other primary or secondary kidney disorders leading to chronic kidney dysfunction.

##### 2.1.1.3 Comparator

Adults without CKD or comparison across different CKD stages. Studies may compare HRQoL between CKD patients and healthy controls, between different stages of CKD, or between different treatment modalities (e.g., hemodialysis versus peritoneal dialysis versus transplantation). Studies without a comparison group but reporting HRQoL data for CKD patients will also be considered if they provide sufficient information to address the review objectives.

##### 2.1.1.4 Outcomes

HRQoL assessed using validated instruments, e.g., a 36-question survey that measures health-related quality of life. It’s used to assess health status in a variety of settings, including clinical practice, research, and population surveys (SF-36), and questionnaires used to measure quality of life (EQ-5D, KDQOL). EQ-5D is a generic instrument that assesses general health, while KDQOL is a disease-specific questionnaire that assesses kidney disease-related quality of life (KDQOL). The primary outcome will be overall HRQoL scores or domain-specific scores (physical, mental, social functioning) as measured by these instruments. Both cross-sectional assessments and longitudinal changes in HRQoL will be considered relevant outcomes.

##### 2.1.1.5 Study design

Observational studies (cross-sectional, cohort, and case-control) and interventional studies. This broad inclusion of study designs acknowledges the diverse methodological approaches used to investigate HRQoL in CKD patients. Observational studies provide valuable real-world evidence about the relationship between CKD and quality of life, while interventional studies may offer insights into how various treatments or management approaches affect HRQoL outcomes.

#### 2.1.2 Exclusion criteria

Studies focusing on pediatric populations, case reports, reviews, and non-English publications will be excluded from this systematic review. Pediatric populations are excluded because children and adolescents with CKD face distinct developmental and psychosocial challenges that differ from those experienced by adults, warranting separate analysis. Case reports are excluded due to their limited generalizability and potential for selection bias. Reviews (narrative, systematic, or meta-analyses) will be excluded to prevent duplication of data, although their reference lists may be screened to identify additional primary studies.

##### 2.1.2.1 Language bias consideration

Non-English publications will be excluded due to resource constraints for translation. We acknowledge this as a potential limitation that may introduce language bias, as relevant studies published in other languages (particularly from non-English speaking countries with high CKD burden) may be missed. This could limit the generalizability of findings, particularly regarding HRQoL experiences in diverse cultural contexts. The potential impact of this exclusion on review findings will be discussed transparently in the final manuscript, and we will compare our findings with any available non-English systematic reviews to assess potential gaps.

### 2.2 Information sources

A comprehensive search will be conducted across PubMed, Embase, Scopus, Web of Science, and Cochrane Library. Grey literature will be searched through ProQuest Dissertations and relevant conference proceedings. For grey literature sources, quality assessment will follow the same Newcastle-Ottawa Scale criteria used for published observational studies, with particular attention to methodological rigor, sample representativeness, and outcome measurement validity. Conference proceedings will be included only if they provide sufficient methodological detail and outcome data to permit quality assessment.

To ensure sensitivity and comprehensiveness, no time limits will be applied. The search will cover publications from database inception through December 2025. If more than six months elapse between the initial search and final review completion, the search will be updated to ensure recently published studies are included.

### 2.3 Search strategy

The search strategy will include terms such as “chronic kidney disease,” “End-Stage Renal Disease,” “Health-Related Quality of Life,” “HRQoL,” “Dialysis,” and “Kidney Transplantation.” Keywords include: adults, patients, quality of life, HRQoL, kidney disease, renal failure, and dialysis, combined using the Boolean operator AND.

#### 2.3.1 Database-specific search algorithms

PubMed: [(QUALITY OF LIFE [Title/Abstract]) AND ADULT [Title/Abstract]) AND KIDNEY DISEASE [Title/Abstract]].

Embase: [(ab: (QUALITY OF LIFE)) AND (ab: (ADULT PATIENTS)) AND (ab: (RENAL FAILURE))].

Scopus: [TITLE-ABS-KEY (quality of life AND adult patients AND dialysis)].

Web of Science: [Title, abstract, keywords: quality of life AND adult patients AND kidney disease].

Cochrane Library: [ab (quality of life) AND ab (adult patients) AND ab (dialysis)].

Google Scholar: To manage the volume of results from Google Scholar, we will limit screening to the first 200 most relevant results ranked by relevance. The search will use: [allintitle: Adult quality of life kidney disease]. This pragmatic approach balances comprehensiveness with feasibility, as Google Scholar searches can yield thousands of results with diminishing relevance.

Grey Literature: ProQuest Dissertations and conference proceedings will be searched using similar keyword combinations, with screening limited to studies from the past 10 years to ensure relevance and feasibility.

Detailed search algorithms for all databases are provided in Appendix A.

### 2.4 Study records

#### 2.4.1 Data management

The systematic review will employ a comprehensive approach to data management to ensure methodological rigor and transparency. All identified studies from database searches will be imported into EndNote X9 reference management software for systematic organization and duplicate removal. Following initial screening, potentially eligible studies will be transferred to a standardized data extraction form developed in Microsoft Excel. This form will be piloted on a sample of five studies to ensure its utility and comprehensiveness before full implementation. The Excel database will be organized with separate worksheets for study characteristics, participant demographics, outcome measures, and quality assessment scores.

To ensure data security and integrity, all files will be stored on password-protected devices with regular backups to cloud storage systems. A clear file naming convention will be established to maintain organization throughout the review process. Each study will be assigned a unique identification code to facilitate tracking and cross-referencing between different aspects of the review. Version control measures will be implemented for all working documents, particularly for the data extraction form, to maintain a clear audit trail of any modifications made during the review process.

#### 2.4.2 Selection process

Two independent reviewers (WWA and VLD) will screen titles and abstracts based on inclusion criteria. Full-text articles of eligible studies will be retrieved and reviewed independently by both reviewers. Discrepancies will be resolved through the following structured process: (1) initial discussion between the two primary reviewers to reach consensus; (2) if consensus cannot be achieved after discussion, detailed documentation of the disagreement; (3) consultation with the third reviewer (CIS) who will make the final determination based on the eligibility criteria and documented rationale from both reviewers. All exclusion decisions and reasons will be documented in an Excel tracking sheet to ensure transparency and reproducibility.

The selection process will follow a systematic, two-stage approach to minimize selection bias. In the first stage, titles and abstracts of all identified records will be screened against predetermined inclusion criteria. Studies that clearly do not meet these criteria will be excluded, while all potentially relevant studies will proceed to full-text assessment.

A PRISMA flow diagram will be generated to document the selection process, detailing the number of studies identified, screened, assessed for eligibility, and included in the final review, with specific reasons for exclusions noted at each stage. Inter-rater agreement between reviewers will be calculated using Cohen’s Kappa coefficient, with values above 0.7 considered indicative of substantial agreement.

A PRISMA flow diagram will be developed and included in the final systematic review manuscript to document the study selection process, including numbers of records identified, screened, excluded (with reasons), and finally included. This diagram is not included in the protocol but will be generated during the review process.

#### 2.4.3 Data collection process

A standardized data extraction form will be used to collect data on study characteristics, population demographics, CKD stage, HRQoL measures, and key findings. Data will be extracted by two independent reviewers.

The data extraction form will be designed specifically for this review, encompassing all relevant variables as outlined in the data items section. Prior to the full data extraction, the form will be piloted on a subset of five included studies and refined based on feedback to ensure its comprehensiveness and usability.

The data extraction form will capture detailed information on study characteristics, population demographics, CKD stage and treatment modalities, HRQoL measurement instruments, outcomes reported, and key findings related to the relationship between CKD and HRQoL. For studies reporting multiple HRQoL measures or time points, all relevant data will be extracted to provide a comprehensive assessment. When encountering missing or unclear information, the review team will attempt to contact the corresponding authors of the original studies via email, allowing a four-week response period before proceeding with analysis of the available data.

Regular calibration exercises will be conducted throughout the data extraction process to ensure consistency between reviewers. Any discrepancies in extracted data will be identified through comparison of the independently completed forms and resolved through discussion between the two reviewers. In cases where agreement cannot be reached, the third reviewer will mediate to determine the final data to be included. This systematic approach to data collection will ensure the accuracy and reliability of the information used for subsequent analysis and synthesis, strengthening the validity of the review findings.

### 2.5 Data items

The following data items will be extracted from each included study:

1. Study characteristics: Author(s), year of publication, country of origin, study design, study setting.
2. Population characteristics: Sample size, age range, gender distribution, CKD stage or ESRD status, comorbidities.
3. CKD-related clinical factors: CKD stage, duration of disease, treatment modality, laboratory values (eGFR, creatinine, hemoglobin).
4. HRQoL domains most affected by CKD: Physical health, mental health, social functioning, psychological health.
5. Treatment-related factors: Type of renal replacement therapy, duration of treatment, frequency of dialysis.
6. Factors influencing HRQoL in CKD patients: Although medication adherence, dietary management, and fluid management are not direct HRQoL outcomes, these variables will be extracted when reported because they represent important behavioral and self-management factors that may mediate or moderate the relationship between CKD and HRQoL. Understanding these factors is essential for developing comprehensive interventions that address both HRQoL outcomes and the self-management challenges that influence them. When these variables are reported in included studies, we will analyze their associations with HRQoL to provide insights for clinical practice.
7. Study limitations and author recommendations.

### 2.6 Outcomes and prioritization

The primary outcome of this systematic review will be health-related quality of life (HRQoL) in adults with chronic kidney disease (CKD) across various stages, including end-stage renal disease (ESRD). HRQoL represents a multidimensional construct encompassing physical, psychological, and social domains of health from the patient’s perspective, extending beyond mere absence of disease or infirmity. This patient-reported outcome has gained increasing recognition as a critical indicator of treatment effectiveness and overall disease burden, complementing traditional clinical endpoints such as mortality and morbidity statistics.

For this review, we will prioritize studies utilizing validated instruments for HRQoL assessment, particularly those widely employed in nephrology research. The 36-Item Short Form Health Survey (SF-36) will be given primary consideration as it measures eight health domains: physical functioning, role limitations due to physical health, bodily pain, general health perceptions, vitality, social functioning, role limitations due to emotional problems, and mental health (3). The Kidney Disease Quality of Life instrument (KDQOL), which combines kidney disease-specific concerns with SF-36 components, will also be prioritized due to its disease-specific relevance and widespread use in CKD research (6). Other validated instruments such as the EuroQol-5D (EQ-5D) will be included to ensure comprehensive coverage of available evidence.

In our analysis, we will examine HRQoL outcomes across multiple dimensions. Physical health domains, including physical functioning, bodily pain, and role limitations due to physical problems, will be assessed to understand CKD’s impact on patients’ physical wellbeing and daily activities. Mental health dimensions, encompassing emotional well-being, psychological distress, and cognitive function, will be evaluated to capture the psychological burden of CKD. Social functioning aspects, including social relationships, role fulfillment, and participation in community activities, will be examined to understand how CKD affects patients’ social interactions and integration. Additionally, disease-specific concerns such as symptoms, treatment burden, and effects of kidney disease on daily life will be prioritized when available from kidney disease-specific instruments.

When analyzing these outcomes, we will give precedence to comparative data that illustrate differences in HRQoL across distinct CKD stages to elucidate the progressive impact of declining kidney function on patient wellbeing. Comparisons between different renal replacement therapies (hemodialysis, peritoneal dialysis, and kidney transplantation) will be prioritized to understand how treatment modalities differentially affect quality of life, potentially informing clinical decision-making regarding treatment options (8, 9). Studies examining relationships between HRQoL and demographic or clinical factors (e.g., age, gender, comorbidities) will be given special attention to identify potential moderators of CKD’s impact on quality of life.

### 2.7 Risk and bias in individual studies

The Newcastle-Ottawa Scale (NOS) will be used for observational studies, and the Cochrane Risk of Bias Tool will be used for interventional studies. Two reviewers will assess bias independently (WWA and VLD). Conflicts will be resolved through discussion between the two reviewers. If consensus cannot be reached, CIS will serve as the third reviewer to make final determinations. All disagreements and final decisions will be documented to ensure transparency.

The Newcastle-Ottawa Scale was selected for observational studies due to its wide acceptance and validated approach to assessing quality in non-randomized studies. This scale evaluates studies across three key domains: selection of study groups, comparability of groups, and ascertainment of exposure or outcome. For cohort studies, the scale assesses representativeness of the exposed cohort, selection of the non-exposed cohort, ascertainment of exposure, demonstration that the outcome of interest was not present at the start of the study, comparability of cohorts, assessment of outcome, adequacy of follow-up duration, and completeness of follow-up. For case-control studies, it evaluates case definition, representativeness of cases, selection of controls, definition of controls, comparability of cases and controls, ascertainment of exposure, same method of ascertainment for cases and controls, and non-response rate.

For interventional studies, the Cochrane Risk of Bias Tool provides a structured approach to evaluating potential sources of bias. This tool assesses random sequence generation, allocation concealment, blinding of participants and personnel, blinding of outcome assessment, incomplete outcome data, selective reporting, and other sources of bias. Each domain will be rated as having low, high, or unclear risk of bias, with detailed justification provided for each assessment.

Two reviewers will independently assess the risk of bias for each included study. Discrepancies in assessments will be resolved through discussion between the reviewers, with consultation of a third reviewer if necessary. The results of these quality assessments will be presented in tabular form and considered in the interpretation of findings. Studies with high risk of bias will not be automatically excluded but will be flagged for sensitivity analyses to determine their influence on overall results. If meta-analysis is conducted, subgroup analyses based on study quality may be performed to explore whether methodological limitations affect the observed relationships between CKD and HRQoL.

### 2.8 Data analysis

Meta-analysis will be performed if sufficient homogeneous data are available. A random-effects model will be used to account for heterogeneity. Heterogeneity will be assessed using the I² statistic. Subgroup analyses will be conducted based on CKD stage, dialysis status, and HRQoL domains.

#### 2.8.1 Handling multiple comparisons

Given the planned subgroup analyses across CKD stages, treatment modalities, HRQoL domains, and demographic factors, we will address the risk of false-positive findings from multiple comparisons using the following approach: (1) Primary analyses will be clearly distinguished from exploratory subgroup analyses; (2) For planned subgroup analyses, we will apply Bonferroni correction when conducting multiple statistical tests within the same outcome domain; (3) P-values will be interpreted with caution, and effect sizes with confidence intervals will be emphasized over significance testing alone; (4) Findings from subgroup analyses will be presented as hypothesis-generating rather than confirmatory, particularly when not pre-specified; (5) Sensitivity analyses will assess the robustness of significant findings.

Publication bias will be evaluated using funnel plots and Egger’s test.

The meta-analysis will employ a random-effects model to synthesize the quantitative findings from included studies, recognizing the expected clinical and methodological heterogeneity across studies of CKD and HRQoL. This approach acknowledges that the true effect size may vary between studies due to differences in study populations, CKD etiology, treatment approaches, and measurement instruments. For continuous outcomes, such as HRQoL scores, mean differences or standardized mean differences will be calculated with 95% confidence intervals, depending on the consistency of measurement scales across studies. When studies use different instruments to measure the same HRQoL construct, standardized mean differences will be employed to facilitate comparability.

Heterogeneity will be formally assessed using the I² statistic, which quantifies the percentage of total variation across studies attributable to heterogeneity rather than chance. I² values of 25%, 50%, and 75% will be interpreted as representing low, moderate, and high levels of heterogeneity, respectively. The chi-squared test will provide a formal statistical test of heterogeneity, with p < 0.10 considered indicative of significant heterogeneity due to the test’s known low power. If substantial heterogeneity is detected (I² > 50%), potential sources will be explored through subgroup analyses and meta-regression if sufficient studies are available.

Planned subgroup analyses include stratification by CKD stage (early versus advanced), treatment modality (conservative management, hemodialysis, peritoneal dialysis, transplantation), demographic factors (age groups, gender), and HRQoL domains (physical, mental, social functioning). These analyses aim to identify factors that might modify the relationship between CKD and HRQoL, providing more nuanced insights for clinical practice. Additionally, stratification by study design and quality assessment scores will be conducted to evaluate the robustness of findings across methodological approaches.

Sensitivity analyses will be performed to assess the impact of methodological decisions and study quality on meta-analytic results. These may include restricting analysis to studies with low risk of bias, excluding studies with imputed data, using alternative meta-analytic models (fixed versus random effects), and excluding potential outlier studies. The results of these sensitivity analyses will be reported transparently to inform interpretation of the primary findings.

### 2.9 Meta-bias

Publication bias will be evaluated using visual inspection of funnel plots and formal statistical tests such as Egger’s test of asymmetry. A funnel plot will be created by plotting the effect estimates against their standard errors or sample sizes. Asymmetry in the funnel plot may indicate publication bias, although other factors such as methodological heterogeneity or chance could also contribute to asymmetry. Egger’s test will provide a statistical assessment of funnel plot asymmetry, with p < 0.05 considered indicative of significant asymmetry.

If publication bias is detected, the trim-and-fill method may be employed to estimate adjusted effect sizes accounting for potentially missing studies. Additionally, the fail-safe N method may be used to calculate the number of unpublished null studies that would be needed to render the observed effect non-significant, providing an indication of the robustness of findings to potential publication bias.

Selective outcome reporting within studies will be assessed by comparing the outcomes specified in study protocols or methods sections with those reported in the results. When available, study protocols will be retrieved from clinical trial registries or published protocol papers. Discrepancies between planned and reported outcomes, particularly the omission of pre-specified outcomes, will be noted as potential indicators of selective reporting bias. The impact of selective reporting will be considered when interpreting the body of evidence and assessing the overall confidence in review findings.

### 2.10 Confidence in cumulative evidence

The quality of evidence across studies for each main outcome will be assessed using the GRADE approach. This systematic method evaluates the certainty of evidence based on study design, risk of bias, inconsistency, indirectness, imprecision, publication bias, magnitude of effect, dose-response relationship, and effect of plausible confounding factors.

#### 2.10.1 GRADE assessment process and disagreement resolution

Two reviewers (WWA and VLD) will independently assess the certainty of evidence for each outcome using GRADE. Disagreements in GRADE ratings will be resolved through: (1) structured discussion between the two reviewers, with explicit documentation of reasoning for each rating decision; (2) reference to the GRADE handbook for clarification of rating criteria; (3) consultation with the third reviewer (CIS) if consensus cannot be achieved, who will review the documented rationale and make a final determination based on GRADE guidelines. All GRADE assessment disagreements and resolutions will be documented in the GRADE evidence profile tables to ensure transparency.

#### 2.10.2 Consideration of study design limitations

We acknowledge that most included studies are likely to be observational (cross-sectional or cohort studies) given the nature of the research question. Observational study designs inherently limit causal inference about the relationship between CKD and HRQoL, as they cannot definitively establish causation or exclude all potential confounding factors. This limitation will be explicitly addressed in the GRADE assessment and in the interpretation of findings. We will clearly communicate that observed associations may be influenced by unmeasured confounders and that causal claims should be made cautiously. Despite these limitations, observational studies provide valuable real-world evidence about HRQoL patterns in CKD patients and can identify important associations that inform clinical practice and hypothesis generation for future interventional research.

For each outcome, the body of evidence will be rated as high, moderate, low, or very low quality. Evidence from randomized controlled trials starts as high quality but can be downgraded based on limitations in the above domains. Conversely, evidence from observational studies starts as low quality but can be upgraded based on factors such as large magnitude of effect, dose-response gradient, or if all plausible confounding would reduce the observed effect.

The assessment will begin with the initial rating based on study design, followed by consideration of factors that might decrease the certainty of evidence: risk of bias (from the quality assessment of individual studies), inconsistency (unexplained heterogeneity in results), indirectness (population, intervention, comparator, or outcome differs from those of interest), imprecision (wide confidence intervals or small sample sizes), and publication bias. Factors that might increase the certainty of evidence will also be considered: large magnitude of effect, dose-response gradient, and confounding factors that would reduce the observed effect.

The results of the GRADE assessment will be presented in a summary of findings table, including the number of studies and participants contributing to each outcome, effect estimates with confidence intervals, quality of evidence ratings, and explanations for downgrading or upgrading decisions. This transparent evaluation of the certainty of evidence will guide the interpretation of findings and inform recommendations for clinical practice and future research regarding the impact of CKD on HRQoL.

The quality of evidence will be assessed using the GRADING of Recommendations Assessment, Development, and Evaluation (GRADE) approach. Two reviewers will independently conduct GRADE assessments for each outcome. Disagreements in GRADE ratings will be resolved through structured discussion between reviewers, focusing on specific criteria causing discordance. If consensus cannot be reached after discussion, a third independent reviewer will arbitrate, with the final rating determined by majority decision. All disagreements and resolutions will be documented to maintain transparency in the assessment process.

For each outcome, evidence will be rated as high, moderate, low, or very low quality, beginning with initial ratings based on study design and considering factors that decrease certainty (risk of bias, inconsistency, indirectness, imprecision, publication bias) or increase certainty (large magnitude of effect, dose-response gradient, confounding factors that would reduce observed effects).

### 2.11 Reporting of review findings

Study selection will be documented using a PRISMA flow diagram showing the number of records identified, screened, assessed for eligibility, and included in the final review, with reasons for exclusions at each stage. The PRISMA flow diagram will be generated during the review process and included in the final manuscript to ensure transparent reporting of the systematic search and selection procedures.

## Data Availability

The original contributions presented in the study are included in the article/supplementary material. Further inquiries can be directed to the corresponding author.

## References

[1] Bowling CB Vandenberg AE Phillips LS Mcclellan WM Ii TMJ Echt KV . Article older patients ’ Perspectives on managing complexity in CKD self-management. Clin J Am Soc Nephrol (CJASN). (2017) 12(4):635–43. doi: 10.2215/CJN.06850616, PMID: 28389529 PMC5383384

[2] Chironda G Bhengu B . Barriers to management of Chronic Kidney Disease (CKD) CKD in a renal clinic in KwaZulu-Natal Province, South Africa – A qualitative study. Int J Afr Nurs Sci. (2019) 10:116–23. doi: 10.1016/j.ijans.2019.04.001

[3] Davey CH Webel AR Sehgal AR Voss JG Huml A . Fatigue in individuals with end stage renal disease. Nephrol Nurs Journal: J Am Nephrol Nurses’ Assoc. (2019) 46:497–508. PMC7047987 31566345

[4] Donald M Kahlon BK Beanlands H Straus S Ronksley P Herrington G . Self-management interventions for adults with chronic kidney disease: A scoping review. BMJ Open. (2018) 8:e019814. doi: 10.1136/bmjopen-2017-019814, PMID: 29567848 PMC5875600

[5] Boima V Dwomoa A Osafo C Tannor E Awuku AY Plange-Rhule J . SAT-017 end stage renal disease in Ghana. Kidney Int Rep. (2019) 4:S8. doi: 10.1016/j.ekir.2019.05.039

[6] Bagasha P Leng M Katabira E Petrova M . Health-related quality of life, palliative care needs and 12-month survival among patients with end stage renal disease in Uganda: protocol for a mixed methods longitudinal study. BMC Nephrol. (2020) 21:541. doi: 10.1186/s12882-020-02197-7, PMID: 33287725 PMC7720495

[7] Luyckx VA Martin DE Moosa MR Bello AK Bellorin-Font E Chan TM . Developing the ethical framework of end-stage kidney disease care: from practice to policy. Kidney Int Suppl. (2020) 10:e72–7. doi: 10.1016/j.kisu.2019.11.003, PMID: 32149011 PMC7031685

[8] Jankowska-Polańska B Uchmanowicz I Wysocka A Uchmanowicz B Lomper K Fal AM . Factors affecting the quality of life of chronic dialysis patients. Eur J Public Health. (2017) 27:262–7. doi: 10.1093/eurpub/ckw193, PMID: 28339523

[9] Jayanti A Foden P Wearden A Mitra S . Illness beliefs in end stage renal disease and associations with self-care modality choice. PLoS One. (2016) 11(7):e0158942. doi: 10.5061/dryad.rg8hj, PMID: 27368055 PMC4930164

[10] Tannor EK Awuku YA Boima V Antwi S . The geographical distribution of dialysis services in Ghana. Renal Replacement Therapy. (2018) 4:14. doi: 10.1186/s41100-018-0143-1

[11] Peng S He J Huang J Lun L Zeng J Zeng S . Self-management interventions for chronic kidney disease: A systematic review and meta-analysis. BMC Nephrol. (2019) 20:142. doi: 10.1186/s12882-019-1309-y, PMID: 31027481 PMC6486699

[12] Mbeje PN Mtshali N Africa S Mbeje P . Perceived predictors of quality of life in patients with end-stage renal disease on dialysis. Curationis. (2021) 44(1):e1–e11. doi: 10.4102/curationis.v44i1.2251, PMID: 34636621 PMC8517801

[13] Adjei DN Adu D Quayson SE Kardaun JWPF Erskine IJ Lartey IS . 20 year trends in renal disease mortality in Ghana: A review of autopsies. Nephrology. (2019) 24:387–94. doi: 10.1111/nep.13255, PMID: 29575514

[14] Noviana CM Zahra AN . Social support and self-management among end-stage renal disease patients undergoing hemodialysis in Indonesia. J Public Health Res. (2022) 11:45–9. doi: 10.4081/jphr.2021.2733, PMID: 35238191 PMC8941312

[15] Pescud M Rychetnik L Allender S Irving MJ Finegood DT Riley T . From understanding to impactful action: systems thinking for systems change in chronic disease prevention research. Systems. (2021) 9(3):61. doi: 10.3390/systems9030061

[16] Stanifer JW Isenburg MV Chertow GM Anand S . Chronic kidney disease care models in low- and middle-income countries: a systematic review. BMJ Global Health. (2018) 3(3):e000728. doi: 10.1136/bmjgh-2018-000728, PMID: 29629191 PMC5884264

[17] Naicker S Ashuntantang G . End Stage Renal Disease in Sub-Saharan Africa. In: Chronic Kidney Disease in Disadvantaged Populations. London, UK: Academic Press (Elsevier imprint). (2017). doi: 10.1016/B978-0-12-804311-0.00014-5

[18] Feehally J Johnson R . The Kidney and High Blood Pressure. In: Comprehensive Clinical Nephrology, 6th ed. Philadelphia, PA: Elsevier. (2018).

[19] KBTH . 2016 annual report. Ghana Health Service Rep. (2016), 1–129.

